# MyD88 Death-Domain Oligomerization Determines Myddosome Architecture: Implications for Toll-like Receptor Signaling

**DOI:** 10.1016/j.str.2020.01.003

**Published:** 2020-03-03

**Authors:** Martin C. Moncrieffe, Daniel Bollschweiler, Bing Li, Pawel A. Penczek, Lee Hopkins, Clare E. Bryant, David Klenerman, Nicholas J. Gay

**Affiliations:** 1 Department of Biochemistry, University of Cambridge, Cambridge CB2 1GA, UK; 2 Department of Chemistry, University of Cambridge, Cambridge CB2 1EW, UK; 3 Department of Biochemistry & Molecular Biology, The University of Texas, McGovern Medical School, Houston, TX 77030, USA; 4 Department of Veterinary Medicine, University of Cambridge, Cambridge CB3 0ES, UK

**Keywords:** TLR signaling, MyD88, Myddosome, IRAK, cryo-EM, TIRFM, light-sheet microscopy

## Abstract

Toll-like receptors (TLRs) are pivotal in triggering the innate immune response to pathogen infection. Ligand binding induces receptor dimerization which facilitates the recruitment of other post-receptor signal transducers into a complex signalosome, the Myddosome. Central to this process is Myeloid differentiation primary response 88 (MyD88), which is required by almost all TLRs, and signaling is thought to proceed via the stepwise, sequential assembly of individual components. Here, we show that the death domains of human MyD88 spontaneously and reversibly associate to form helical filaments *in vitro*. A 3.1-Å cryoelectron microscopy structure reveals that the architecture of the filament is identical to that of the 6:4 MyD88-IRAK4-IRAK2 hetero-oligomeric Myddosome. Additionally, the death domain of IRAK4 interacts with the filaments to reconstitute the non-stoichiometric 6:4 MyD88-IRAK4 complex. Together, these data suggest that intracellularly, the MyD88 scaffold may be pre-formed and poised for recruitment of IRAKs on receptor activation and TIR engagement.

## Highlights

• MyD88^DD^ self-associates *in vitro* to form helical filaments
• Filament architecture is identical to that of the Myddosome
• IRAK4 interacts with filaments to reconstitute 6-MyD88:4-IRAK4 complex
• A MyD88 pre-Myddosome scaffold exists *in vivo*

## Teaser

Moncrieffe et al. describe the 3.1-Å cryo-EM structure of MyD88^DD^ filaments, which have the same helical architecture as the Myddosome and interact with IRAK4^DD^*in vitro*. They present evidence of a pre-signaling hexameric MyD88 scaffold *in vivo* and propose an inhibitory role for the TIR domain.

## Introduction

Toll-like receptors (TLRs) are pattern-recognition receptors of the innate immune system that are activated when conserved molecular signatures on microbial or host molecules are detected (Akira et al., 2006, Seong and Matzinger, 2004). Architecturally, TLRs possess an extracellular ligand binding domain composed of leucine-rich repeats (LRRs), a single transmembrane segment, and a cytosolic signaling domain that bears homology to the *Drosophila melanogaster* protein Toll and interleukin-1 receptor (TIR) (Gay and Keith, 1991). A diverse number of disease states have been implicated with dysfunction in TLR signaling, including autoimmune disorders (Fischer and Ehlers, 2008), inflammation (Joosten et al., 2016), and cancer (Chen et al., 2008).

The human genome encodes ten TLRs and all, with the exception of TLR3 (Akira et al., 2006), require the cytosolic adaptor human Myeloid differentiation primary response 88 (hMyD88). Signal transduction is thought to proceed sequentially by the stepwise assembly of proteins involved in the cascade initiated by ligand binding to the LRRs on the receptor ectodomain, which leads to the formation of an activated receptor dimer. Receptor ectodomain dimerization facilitates association of its cytosolic TIR domains, providing a scaffold for the recruitment of other TIR-containing adaptor proteins, in particular, hMyD88 and MAL/TIRAP (Akira et al., 2006, Gay et al., 2014). hMyD88 also encodes an N-terminal death domain, which is joined to the TIR by a linker region, the intermediate domain. *In vitro*, hMyD88 death domains (hMyD88^DD^) assemble with the homologous domain on the interleukin-1 receptor-associated kinases (IRAKs) to produce an oligomeric structure, the Myddosome, which has an unusual stoichiometry of 6–8 hMyD88 subunits and precisely four each of IRAK4 and IRAK2 (Lin et al., 2010, Motshwene et al., 2009). A crystal structure of this complex reveals a left-handed helical arrangement of death domains in which hMyD88^DD^ forms two layers, one of which interacts with IRAK4. IRAK2 then completes the ternary complex by associating with IRAK4 (Lin et al., 2010). *In vivo* single-molecule imaging data suggest that activated TLR receptor dimers nucleate the rapid assembly and disassembly of a Myddosome complex similar to that observed *in vitro* (Latty et al., 2018).

Several TLRs require phosphatidylinositol 4,5-bisphosphate binding (Kagan and Medzhitov, 2006), and TIR-containing, membrane-localized adaptor protein MAL/TIRAP for optimal signaling. A possible template for how interactions between receptor and adaptor TIRs nucleate Myddosome assembly has been obtained from the structure of the helical MAL^TIR^ filament (Ve et al., 2017), which is assembled from protofilaments composed of parallel strands of MAL^TIR^ subunits held together by intra- and inter-strand interactions. In this model, the cytosolic TIR scaffold is helical and consists of intra-strand contacts between receptor TIRs. Adaptor TIRs, for example, those of hMyD88^TIR^, are recruited sequentially to the receptor TIR scaffold by inter-strand contacts (Ve et al., 2017) followed by the recruitment of IRAK4 to hMyD88 via death-domain interaction and subsequently by other members of the IRAK family whose phosphorylation drives downstream signaling events.

The stepwise sequential model is attractive because it explains known biochemical data and presents information flow during TLR signaling as a linear sequence of events whereby signal propagation depends on the assembly of components from the preceding step. However, the model does not explain, for example, how the unusual stoichiometry of the Myddosome would arise by a stepwise assembly process. Additionally, the TIR domain of hMyD88 is envisaged to act as a nucleating hub (Latty et al., 2018, Ve et al., 2017, Vyncke et al., 2016), which facilitates the oligomerization of hMyD88^DD^ observed in the Myddosome (Lin et al., 2010, Motshwene et al., 2009).

In this work we show that the scaffold for the Myddosome created by the oligomerization of hMyD88^DD^ can exist as a pre-formed structure *in vivo* and suggest an alternative path for Myddosome assembly in which receptor or adaptor TIR engagement with the homologous domain in hMyD88 provides the switch that allows IRAK4 recruitment with the death-domain scaffold and further downstream signal transduction.

## Results

### hMyD88^DD^ Spontaneously Assembles into Helical Oligomers

**Figure 1:**
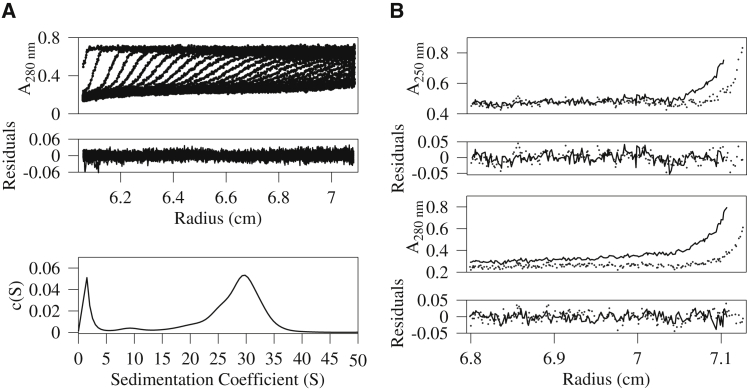
Sedimentation Velocity and Equilibrium Measurement of the Homo-oligomeric MyD88 Death-Domain Complex (A) Velocity data, residuals after fitting, and the c(S) distribution for the hMyD88 death-domain complex. The c(S) distribution is broad and has a peak at 30 S. (B) Sedimentation equilibrium data recorded at 250 nm and 280 nm and rotor speeds of 3,000 and 4,000 rpm. The recovered molecular weight of the hMyD88^DD^ oligomer from a global analysis of these data was 5.2 MDa.

Solutions of hMyD88^DD^ form oligomers at concentrations above 23 μM (Motshwene et al., 2009). Circular dichroism (CD) in the far UV, performed on samples of hMyD88^DD^ at concentrations of 35 μM and higher, displayed characteristic spectral bands at 209 nm and 223 nm (Figure S1A), which is indicative of α-helical secondary structure. Monomeric hMyD88^DD^ is predominantly helical (Lin et al., 2010), implying that its oligomeric form is also helical and is unlikely to be composed of denatured aggregates. To obtain hydrodynamic data on the nature of the self-associating hMyD88^DD^ oligomer, we acquired sedimentation velocity and sedimentation equilibrium data on samples having concentrations of 35 μM or higher. A c(S) analysis of the sedimentation velocity data suggests that the hMyD88^DD^ oligomer is heterogeneous, having sedimentation coefficients ranging from approximately 23 to 34 S (Figure 1A). There is evidence of monomeric hMyD88^DD^, which sediments at 1.5–2 S (Motshwene et al., 2009), suggesting a dynamic equilibrium between monomeric and oligomeric forms of hMyD88^DD^. The frictional coefficient from this analysis was 1.3 but the very flat error surface for this parameter suggests it was not well determined and, hence, that information regarding the shape or molecular weights from the sedimentation velocity data would be unreliable. The molecular weight recovered from a global analysis of the sedimentation equilibrium data (Figure 1B) collected at two rotor speeds (3,200 and 4,200 rpm) and two wavelengths (250 and 280 nm) was 5.2 MDa. Given the heterogeneous sedimentation velocity data, this likely represents an average molecular weight value. Additionally, because the molar mass of the hMyD88^DD^ monomer is 17.5 kDa, the equilibrium data imply that the helical oligomer is composed of at least several hundred hMyD88^DD^ monomers. The thermal stability of the hMyD88^DD^ helical oligomer was determined by monitoring the fluorescence of SPYRO orange and also by changes in secondary structure at fixed wavelength using CD spectroscopy, both as a function of temperature. Although the measured signal in these methods arises from different sources, the midpoints of the melting profiles are similar, corresponding to T_m_ values of 53.5°C and 55.7°C for the fluorescence and CD measurements, respectively (Figures S1C and S1B). Additionally, the pre-transition slope obtained by monitoring the secondary structure content at fixed wavelength suggests that the hMyD88^DD^ helical oligomer likely dissociates before the melting of the helices in the monomeric protein occurs.

### Cryo-EM Structure of the hMyD88^DD^ Helical Filament

**Figure 2:**
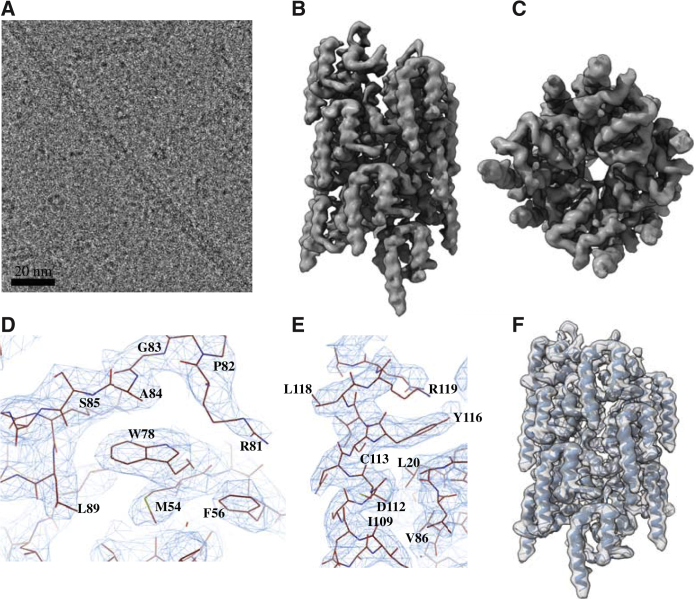
Cryo-EM Structure of the hMyD88^DD^ Filament at 3.1 Å Resolution (A) Typical cryo-EM image of the hMyD88^DD^ filaments, which have lengths ranging from approximately 10 to 1,000 nm. (B–F) Two views of the reconstructed hMyD88^DD^ complex (B and C) with the latter looking down the helical axis and showing the approximately 16-Å cavity that spans the filament. The long helix (H6) at the C terminus of the hMyD88^DD^ is clearly visible. The quality of the cryo-EM map allowed unambiguous fitting of atomic models of hMyD88^DD^ monomers into the reconstructed map. Typical side-chain densities of some residues in the hydrophobic region surrounding W78 (D) and the sixth α-helix, H6 (E). Cartoon representation of a 13-subunit segment of the hMyD88^DD^ monomers superimposed onto the reconstructed cryo-EM map (F).

Having established that hMyD88^DD^ can self-associate into helical assemblies, we sought to determine the precise arrangement of these oligomeric forms. Preliminary negative-stain electron microscopy revealed that hMyD88^DD^ assembled into filaments of varying lengths, with a diameter of approximately 68 Å, and these were corroborated by cryoelectron microscopy (cryo-EM) images (Figure 2A). Subsequent analysis of the cryo-EM data using SPHIRE (Moriya et al., 2017) revealed that the filaments possessed helical symmetry, having an axial rise of approximately 6.2 Å and an azimuthal rotation angle of 98.2°. Iterative helical real-space refinement (Egelman, 2000) as implemented in RELION (He and Scheres, 2017, Scheres, 2012) was used to calculate a 3.1-Å cryo-EM map—as judged by the Fourier shell correlation (FSC) = 0.143 criterion (Figure S3C)—of the hMyD88^DD^ filament with refined helical parameters of 5.98 Å and 98.01°. The computed maps were of excellent quality with elements of secondary structure clearly visible (Figures 2B and 2C). A copy of the hMyD88^DD^ monomer derived from the atomic coordinates of the heterotrimeric MyD88-IRAK4-IRAK2 death-domain complex (Lin et al., 2010) was fitted into the map using MOLREP (Vagin and Isupov, 2001) and the atomic coordinates refined using PHENIX (Adams et al., 2010). Typical fits of the refined side-chain density into the post-processed EM map are shown in Figures 2D and 2E, which clearly illustrate the good agreement between map and model. This enabled model building to extend the N terminus by a single residue and also the C-terminal helix (H6) by three residues relative to the previously deposited model (Lin et al., 2010) and, consequently, extends the death-domain boundary of hMyD88 to Q121. The root-mean-square deviation (RMSD) between a MyD88^DD^ subunit taken from the filament and that from the crystal structure of the Myddosome (Lin et al., 2010) was 0.76 Å. Figure 2F shows the refined coordinates of 13 hMyD88^DD^ subunits superimposed on the cryo-EM map. The tower ^DD^-shaped layered structure is reminiscent of the ternary hMyD88-IRAK4-IRAK2 complex (Lin et al., 2010). Interestingly, although the hMyD88^DD^ construct used has a 30-residue segment belonging to the intermediate domain (ID), which links the death and TIR domains, this region is absent from the reconstructed maps, suggesting that it lacks ordered secondary structure. This suggests that one role of the ID is to act as a flexible tether that allows the TIR and death domains of hMyD88 to adopt a range of orientations with respect to each other. In addition, it implies that the ID plays no role in the self-association of hMyD88^DD^. Parameters relating to cryo-EM data collection, refinement, and validation are summarized in Table 1.

**Table 1:** Cryo-EM Data Collection, Refinement, and Validation Statistics

	hMyD88^DD^

**Data Collection**	

Microscope	Titan Krios
Camera	Falcon III
Operating voltage (kV)	300
Magnification	75,000 ± 1,500
Dose per frame (e/Å^2^)	0.92
Pixel size (Å)	1.06
Defocus (μm)	−0.9 to −3.2

**Reconstruction**	

Overlapping helical segments	1,229,488
Point group symmetry	C_1_
Refined helical symmetry	5.98 Å; 98.01°
Resolution (Å)	3.1
Resolution measure	Map FSC
Map-sharpening *B* factor (Å^2^)	−150

**Model Composition**	

Monomer (protein residues; non-hydrogen atoms)	103; 2,206
13-mer (protein residues; non-hydrogen atoms)	1,339; 10,660

**Refinement and Validation (Monomer; 13-mer)**	

RMS bond length (Å)	0.005; 0.007
RMS angles (°)	0.94; 1.041
Rotamer outliers (%)	0; 0
Ramachandran favored (%)	95.05; 92.08
Ramachandran allowed (%)	4.95; 7.92
MolProbity score	1.44; 1.64
Clashscore	3.02; 3.63

### Inter-subunit Interactions Stabilize Filament Formation

**Figure 3:**
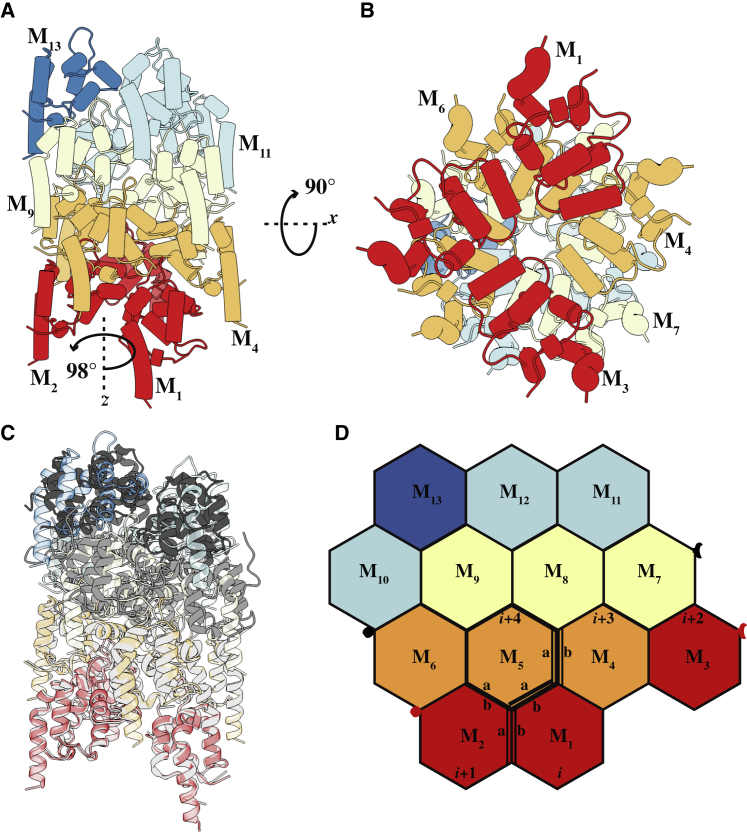
Structure of the hMyD88^DD^ Filament (A) A segment composed of 13 hMyD88^DD^ subunits (M1–M13). The 13 death-domain subunits are assembled in layers and each layer is colored differently. (B) View along the helical axis of the hMyD88^DD^ filament and some inter-subunit contacts. (C) Superposition of the heterotrimeric hMyD88^DD^ (light gray), IRAK4^DD^ (medium gray), and IRAK2^DD^ (dark gray) Myddosome onto the hMyD88^DD^ filament. The MyD88 layer of the heterotrimer superimposes onto the helical filament with an RMSD of 0.76 Å. (D) Representation of the different interfaces present in the hMyD88^DD^ helical filament. The interaction between the *i*+1 and *i*+4 subunits generates a type I interface while that between the *i* and *i*+4 subunits generates a type II interface. The type III interface, which occurs at the intersection of type I and II interfaces, is represented by the interaction between the *i*+3 and *i*+4 subunits. Red and black clasps indicate where the M_3_ and M_7_ subunits fit when the two-dimensional hexagons are wrapped to represent the helical filament.

The hMyD88^DD^ filament can be described as a single-stranded left-handed helix of death domains and is similar to the ternary death-domain complex (Lin et al., 2010). However, unlike the arrangement in the hMyD88-IRAK4-IRAK2 death-domain complex, each hMyD88^DD^ subunit—with the exception of those at the end of the filaments—resides in an equivalent environment. The architecture of the helical death-domain filament is shown in Figure 3A using a 13-subunit segment. Each individual hMyD88^DD^ monomer makes immediate contact with six neighboring death domains, and this is most clearly seen in Figure 3D where the three-dimensional filament is represented in two dimensions. The inter-subunit interfaces are composed of type I(a/b), type II(a/b), and type III(a/b) interactions (Park et al., 2007, Weber and Vincenz, 2001) (Figure S4). The type I interface is predominantly electrostatic in nature and involves interactions between H1 and H4 on one subunit (type Ia) with H2 and H3 of a second subunit. The residues involved in the type I(a/b) interface of the hMyD88^DD^ filament, which is represented by the M_2_-M_5_ interaction (*i*+2 and *i*+4 subunits) (Figure 3C), bury 622 Å^2^. The type II interface, which occurs between the *i* and *i*+4 subunits, for example, is smaller than the type I interface and buries 511 Å^2^. This surface involves the interaction between residues on H4 and the loop connecting the H4 and H5 helices on one subunit (type IIa) with residues on H6 and the H5-H6 loop on a second subunit (type IIb). The type III interface involves interaction between residues on H3 of one subunit (type IIIa) and residues close to the H1-H2 and H3-H4 loops of the second subunit. This surface is typified by the *i*+3 and *i*+4 interaction (Figure 3C) and is the smallest surface, burying only 253 Å^2^. As illustrated in Figure 3D, type I and type II interactions occur between successive layers while the type III interaction occurs within each layer. Therefore, hMyD88^DD^ filament formation likely proceeds via an initial “seed” consisting of a type I or type II homodimer, which then associates into a tetramer and continues to grow. Details of the residues involved in the various interfaces are listed in Table S1. Superposition of the ternary MyD88-IRAK4-IRAK2 death-domain structure on that of the hMyD88^DD^ filament is shown in Figure 3C. The RMSD between MyD88^DD^ from the ternary complex and that from filament is 0.76 Å, confirming the similarity in architecture of both structures. The largest differences occur between the loops connecting the H1-H2, H3-H4, and H4-H5 helices (Figure S5).

### IRAK4^DD^ Interacts with the hMyD88^DD^ Filament

**Figure 4:**
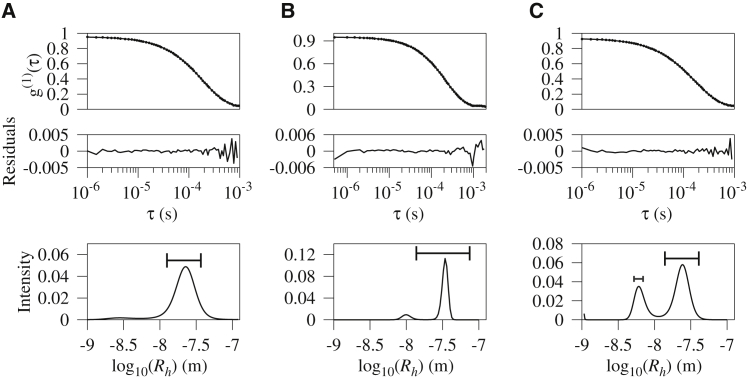
IRAK4^DD^ Associates with the hMyD88^DD^ Filament The upper and middle panels show plots of the autocorrelation as a function of time and residuals after fitting while the bottom panel shows a plot of intensity as a function of hydrodynamic radius for the hMyD88^DD^ death-domain filament at 34 μM (A) and 45 μM (B), with the length of each bar representing the average filament length. At the highest concentration, the complex has a hydrodynamic radius of approximately 31 ± 9 nm and the distribution shifts to larger oligomers. The addition of IRAK4^DD^ death domain (C) results in the formation of the previously characterized hMyD88^DD^-IRAK4^DD^ Myddosome (blue).

A common view of the TLR signal transduction pathway envisages receptor dimerization as the crucial step that facilitates association of TIR domains on both the receptor and the adaptor MyD88. MyD88 subsequently recruits the IRAKs to complete the signaling platform. Given that IRAK4 is indispensable for MyD88-dependent signaling (Suzuki et al., 2002), we sought to determine whether the helical hMyD88^DD^ filament interacts with the death domain of IRAK4. Dynamic light scattering (DLS) was used to measure the size distributions of the hMyD88^DD^ filament at two concentrations and additionally when titrating aliquots of IRAK4^DD^ such that the effective concentration of hMyD88^DD^ was always above that required for filament formation. Because IRAK4 is monomeric, a change in the size distribution from the hMyD88^DD^ filament to the 6–8:4 hMyD88^DD^-IRAK4^DD^ Myddosome should be observable. Figures 4A and 4B shows DLS measurement of hMyD88^DD^ filaments at 34 μM and 45 μM. At 45 μM, the hMyD88^DD^ oligomers are longer, as would be expected from a process whereby filament growth is concentration dependent. The addition of aliquots of IRAK4^DD^ to the hMyD88^DD^ oligomer in Figure 4B enables the formation of the 6:4 hMyD88^DD^-IRAK4^DD^ Myddosome (Figure 4C). This was confirmed by DLS measurements using purified samples of the hMyD88^DD^-IRAK4^DD^ Myddosome (Figure S2B) in addition to sedimentation velocity experiments (Figure S2A), which recovered a sedimentation coefficient value of 6.1 S, similar to the published value (Motshwene et al., 2009). Of note, there is no evidence of small oligomeric species, for example, a heterodimer of hMyD88^DD^ and IRAK4^DD^. Consequently, a mechanism of Myddosome assembly that involves the stepwise dissociation of hMyD88^DD^ monomers from the filament by IRAK4^DD^ and their reconstitution into a 6:4 hMyD88^DD^-IRAK4^DD^ complex is not supported. In contrast, the data support a model in which IRAK4^DD^ monomers associate with one end of the helical filament, resulting in the formation of a transient 4-IRAK4^DD^-hMyD88^DD^ filament and its subsequent dissociation to produce the 6:4 hMyD88^DD^-IRAK4^DD^ Myddosome and a shortened hMyD88^DD^ filament (Figure 4C).

### The Myddosome Scaffold Is Pre-formed *In Vivo*

Overexpression of MyD88 in several cell types results in large cytosolic aggregates of unknown structure and composition (Jaunin et al., 1998, Nishiya et al., 2007). Additionally, using a cell-free expression system that enabled the tuning of protein expression levels, it has been shown that MyD88 polymerizes at low concentrations by a process in which both death and TIR domains are implicated (O'Carroll et al., 2018). Given that hMyD88^DD^ self-associates into helical filaments *in vitro* having symmetry identical to that of the hMyD88^DD^-IRAK4^DD^-IRAK2^DD^ Myddosome (Lin et al., 2010), we sought to determine whether MyD88 oligomerization *in vivo* could produce a cytosolic “pre-Myddosome” scaffold prior to receptor activation. In a previous study (Latty et al., 2018), we used immortalized bone marrow-derived macrophages from MyD88^−/−^ mice that had been reconstituted with a functional, fluorescent form of MyD88 to study Myddosome formation. This experimental system accurately recapitulates the physiological signaling process, as stimulation with lipopolysaccharide (LPS) leads to rapid formation of Myddosomes at the plasma membrane and nuclear factor κB (NF-κB) activation. We have therefore used both total internal reflection fluorescence microscopy (TIRFM) and single-molecule light-sheet imaging to estimate the stoichiometry of MyD88 in unstimulated and LPS-treated cells.

**Figure 5:**
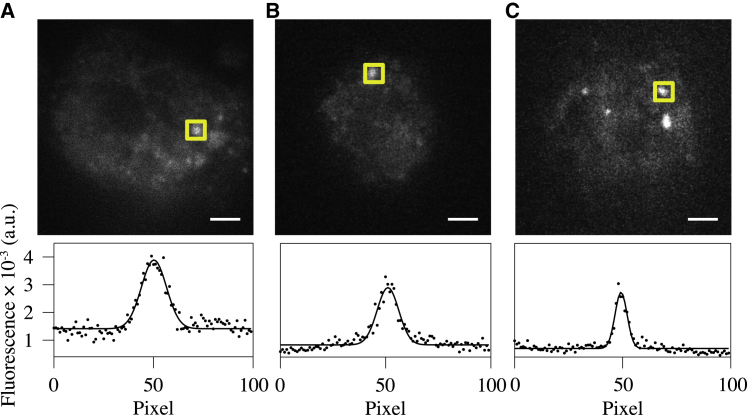
Full-Length MyD88 Self-associates to Produce a Pre-Myddosome Scaffold *In Vivo* (A and B) Representative TIRFM (A) and light-sheet (B) images of GFP-MyD88 expression in unstimulated virally transduced MyD88^−/−^ bone-derived macrophage (n = 31). (C) shows a typical TIRFM image after stimulation with LPS (n = 8). Fitting the intensity distribution of the oligomeric GFP-MyD88 species using a box size of 100 pixels to a Gaussian allows determination of the peak intensity, which is used along with that of monomeric GFP-MyD88 (Figures S6C and S6D) to estimate the number of associating MyD88 monomers. For TIRFM and light-sheet illumination in both unstimulated and LPS-stimulated cells, the estimated number of MyD88 monomers was 1 and 5 ± 1, respectively. The scale bars represent 5 μm.

Figure 5 shows TIRFM and light-sheet fluorescence microscopy (LSFM) images of GFP-MyD88 expression in unstimulated cells (Figures 5A and 5B) and the corresponding TIRFM (Figure 5C) image of cells stimulated with LPS. Analysis of the intensity distribution (Figures S6A and S6B) in unstimulated cells by both methods (Ponjavic et al., 2018) reveals the presence of monomeric, dimeric, and hexameric populations of GFP-MyD88. The average number of pre-formed hexamers obtained using LSFM from 31 unstimulated cells was one, while cells stimulated with LPS typically contained three to four MyD88 hexamers in a given imaging volume. Given that signaling is not observed in unstimulated cells (Latty et al., 2018), these data imply that unstimulated cells contain a small population of hexameric MyD88 oligomers whose conformation does not allow recruitment of IRAKs.

## Discussion

Activation of TLRs initiates a signaling cascade that culminates in the production of pro-inflammatory molecules. The commonly accepted view holds that the initial event involves ligand binding to LRR motifs on the receptor ectodomain. This creates activated homo- or heterodimeric receptor complexes whose cytosolic TIR domains engage with other TIR-containing adaptor proteins including hMyD88 and the subsequent formation of the Myddosome (Lin et al., 2010, Motshwene et al., 2009), which constitutes the core of the bowtie (Beutler, 2004, Friedlander et al., 2015) signaling network for all TLRs except TLR3. While interactions between the TIR domains of TLRs and adaptors and also TIR-containing adaptors have long been implicated, direct evidence *in vitro* has been sparse. However, recently the TIR domain of MAL/TIRAP was found to form open-ended homo-oligomeric helical filaments and, additionally, hetero-oligomeric filaments with the homologous domain of hMyD88 via a two-stranded head-to-tail arrangement (Ve et al., 2017). Assembly of the MAL^TIR^ filament involves intra- and inter-strand interactions, and this has led to a model of TLR signaling in which receptor dimerization on ligand binding leads to intra-strand contacts between receptor TIR domains. Subsequent inter-strand interaction between the newly created receptor TIR dimer interface with other TIR-containing adaptors such as MAL^TIR^ or hMyD88^TIR^ then occurs, leading to the formation of higher-order receptor-associated complexes (Ve et al., 2017).

The aforementioned suggests a stepwise, sequential mechanism for the assembly of receptor and adaptor TIRs and, by extension, the Myddosome. Additionally, it implies that oligomerization of hMyD88 is driven, at least in part, by receptor TIR-hMyD88^TIR^ or MAL^TIR^-hMyD88^TIR^ interactions. However, our finding that hMyD88^DD^ spontaneously forms helical filaments whose architecture is identical to that observed in the heterotrimeric hMyD88-IRAK4-IRAK2 death-domain complex (Lin et al., 2010) suggests that the helical arrangement adopted by hMyD88 during signaling is an intrinsic property of its death domain and is therefore independent of its interaction with either receptor or adaptor TIRs. Given that these filaments also interact with IRAK4^DD^ to form the previously characterized hMyD88^DD^-IRAK4^DD^ complex (Motshwene et al., 2009), it also implies that the hMyD88^DD^ scaffold may not assemble sequentially during signaling but may exist, intracellularly, as a pre-formed structure. Overexpression of full-length MyD88 in HEK293T, bone marrow-derived macrophages, or HeLa cells resulted in large species scattered throughout the cytosol having sizes that are in the micron range (Jaunin et al., 1998, Nishiya et al., 2007). We contend that these previously uncharacterized forms of hMyD88 are in fact helical filaments assembled from hMyD88^DD^ interactions as described. The physiological role of these filaments is unknown, but our finding that MyD88^DD^ alone self-assembles implies that “super-Myddosomes,” which contain more than six copies of MyD88, are possible, and additionally prompted us to look for oligomeric forms of MyD88 in unstimulated cells. Given that filament formation and length is concentration dependent, one expects that in cellular contexts where hMyD88 expression is normal, the oligomeric helical forms will attain shorter lengths than those observed when the protein is overexpressed.

This is consistent with our finding that hexameric oligomers of full-length hMyD88 exist in unstimulated macrophage cells and with recent reports suggesting that the polymerization of full-length MyD88 occurs at concentrations in the nanomolar range by a process that requires the death and TIR domains (O'Carroll et al., 2018).

**Figure 6:**
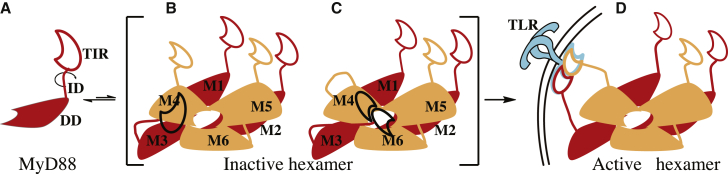
Model of TLR Signaling in which the TIR Domain of hMyD88 Acts as a Binary Switch (A–D) (A) MyD88 contains an N-terminal death domain (DD) and a C-terminal TIR domain, which are linked by an intermediate domain (ID) that is devoid of ordered secondary structure. Cytosolic MyD88 reversibly self-associates via its DD to form an inactive two-layered (M1–M3 and M4–M6) hexameric scaffold that is incapable of producing a signaling Myddosome because the M3–M6 surface, which is required for IRAK4 DD interaction, is obscured by the TIR domain. This inactive conformation could be caused by the TIR domain of the M3 MyD88 subunit (black) from the first layer blocking the M4 and M6 IRAK4 interaction surfaces (B) or, alternatively, weak association between the M3 and M4 TIR domains, for example as shown in (C). Microbe-associated molecular pattern and coreceptor binding—where required—to the receptor ectodomain enable dimerization and activation of receptor TIR domains, which associate directly via intra-strand contacts (D). This receptor TIR dimer interacts strongly with the hMyD88 TIR domain via inter-strand contacts directly or via MAL/TIRAP, and this relieves the inhibition of the TIR domain and allows recruitment of the IRAK4 and IRAK2 onto the hMyD88 scaffold where signal transduction proceeds by phosphorylation.

Models of Myddosome assembly should be consistent with reported signaling data. For example, a truncated hMyD88 construct lacking only the TIR domain elicited NF-κB activity similar to that of the full-length protein (Medzhitov et al., 1998, Wesche et al., 1997). This suggests that the death and intermediate domains are sufficient to recruit IRAKs whose subsequent phosphorylation determines signal flux through TRAF6 and, hence, NF-κB production. It also implies that a key step in Myddosome formation, the oligomerization of hMyD88 death domains, occurs intracellularly without the involvement of the TIR domain of the receptor, other adaptors, or that of hMyD88 itself. Another interesting observation regards MyD88_S_, a splice variant that lacks the ID and inhibits TLR/interleukin-1 receptor (IL-1R) signaling by preventing the recruitment of IRAK4 and, hence, IRAK4-mediated phosphorylation of IRAK1 (Burns et al., 2003). The heterotrimeric Myddosome structure (Lin et al., 2010) reveals that IRAK4^DD^ interacts with only one end of the helical scaffold created by the oligomerization of hMyD88^DD^. Thus, the inability of hMyD88_S_ to associate with IRAK4 could be due to failure of hMyD88_S_^DD^ to self-associate in the absence of the ID or obstruction of the surface required for IRAK4 binding by the TIR domain of hMyD88_S_. The latter explanation is more plausible because the construct used by Lin et al. (2010) lacks the ID but is able to self-oligomerize and associate with IRAK4^DD^. The preceding raises the possibility that the TIR domain of hMyD88 may function as a binary switch during TLR/IL-1R signaling. It has been reported that forced dimers of MyD88^TIR^ interact strongly with activated TLRs (Fekonja et al., 2012). In the absence of receptor or adaptor TIR engagement with hMyD88, its TIR domain exerts an inhibitory effect, thus preventing the recruitment of IRAK4 and subsequent Myddosome formation. Conversely, this inhibition is removed when either activated receptor TIR domains engage directly or indirectly via MAL with the TIR domain of hMyD88. Removal of inhibition by the TIR domain of hMyD88 is facilitated by the ID, which is envisaged to act as a “lever” allowing the TIR domain of hMyD88 to adopt an orientation in which it no longer blocks the surface on the hMyD88^DD^ scaffold required for IRAK4^DD^ engagement and, additionally, positions the TIR domain optimally for interaction with the juxtamembrane TIR domains of the activated receptor or adaptor TIRs. This leads to a second model of TLR signaling, which is summarized in Figure 6. Intracellularly, monomeric hMyD88 exists in equilibrium with low concentrations of short hexameric helical forms that have the same architecture as the Myddosome. However, the TIR domain prevents constitutive pathway activation by obscuring the surface on the MyD88 platform with which IRAK4 interacts. Receptor activation on microbe-associated molecular pattern binding creates the necessary intra-strand receptor dimer as proposed by Ve et al. (2017), and this newly created surface recruits the hMyD88 assembly by homotypic TIR-TIR interactions, which then allows recruitment of the IRAKs to the hMyD88 scaffold. In this model, the hexameric MyD88 platform is envisaged to act as a “sentinel” that can be quickly utilized. Subsequent signaling may then proceed via nucleation as previously described (Gay et al., 2014). Interestingly, a constitutively active mutant of hMyD88, L265P, which has been implicated in several cancers including Waldenström's macroglobulinemia (Ngo et al., 2011, Treon et al., 2012), does not require receptor attachment for signaling (Avbelj et al., 2014). Although the L265P mutation maps to the intra-strand TIR interface (Lin et al., 2010), the non-requirement of receptor TIR engagement for NF-κB production strongly suggests that this mutation is involved in a process that relieves the inhibition of the TIR domain as described.

TLRs respond to a range of ligand concentrations, and this bears resemblance to processes occurring in chemotactic bacteria whose receptors simultaneously detect low concentrations and ligand gradients over several orders of magnitude (Mesibov et al., 1973). A mathematical model suggests that this requirement can be satisfied if both single receptors and clusters of receptors reside on the cell (Bray et al., 1998). Receptor clustering has been observed in TLR4 signaling (Motshwene et al., 2009) and it is likely that other TLRs behave similarly. Additionally, during TLR signaling it is unlikely that only a specific receptor type is activated, given that pathogens will produce a variety of activating ligands. It has been shown that TLR pathway crosstalk enhances cytokine secretion (Lin et al., 2017) and thus different TLR types localized in the same compartment—for example, plasma membrane-bound TLR4 and TLR1/2, which respond to bacterial lipopolysaccharide and lipopeptide—could act synergistically. Whether the TIR domains of hMyD88 from one Myddosome are able to simultaneously interact with the receptor TIR dimer or the MAL-TIR/receptor TIR tetramer from two different TLR receptor types, while plausible, is currently not known.

In summary, this study proposes an additional model for TLR signaling via the Myddosome in which oligomerization of hMyD88 to produce the scaffold required for IRAK4 interaction is produced prior to receptor activation. Constitutive pathway activation is, however, prevented by the TIR domain of hMyD88, which is inhibitory until TIR dimers from this scaffold engage with activated receptor TIR dimers or receptor TIR/MAL-TIR heterotetramers.

## STAR★Methods

### Key Resources Table

**Table:** 

REAGENT or RESOURCE	SOURCE	IDENTIFIER
**Bacterial and Virus Strains**		

E. coli Rosetta 2(DE3)	Merck Millipore	Cat # 71400

**Deposited Data**		

Cryo-EM map and atomic model of MyD88 DD filament	This study	EMD-4405
Atomic model of MyD88 DD filament	This study	PDB 613N
Atomic model of the MyD88-IRAK4-IRAK2 complex	Lin et al., 2010	PDB 3MOP

**Recombinant DNA**		

pMCSG7	DANSU	https://dnasu.org/
p891	Latty et al., 2018	N/A
pHR MyD88 GFP	Latty et al., 2018	N/A
pMDG	Latty et al., 2018	N/A

**Software and Algorithms**		

Sedfit	Schuck, 2000	http://analyticalultracentrifugation.com/
SPHIRE	Moriya et al., 2017	http://sphire.mpg.de
Relion-3.0	Scheres, 2012	https://www3.mrc-lmb.cam.ac.uk/
MotionCor2	Zheng et al., 2017	https://emcore.ucsf.edu/ucsf-motioncor2
Gctf-1.06	Zhang, 2016	https://www.mrc-lmb.cam.ac.uk/kzhang/
MOLREP (CCPEM)	Vagin and Isupov, 2001	http://www.ccpem.ac.uk
UCSF Chimera	Pettersen et al., 2004	https://www.cgl.ucsf.edu/chimera/
COOT	Emsley et al., 2010	https://www2.mrc-lmb.cam.ac.uk/personal/pemsley/coot/
PHENIX	Adams et al., 2010	http://www.phenix-online.org

**Other**		

Spyro Orange	Thermo Fisher	Cat # S6650
StrepTrap HP	GE Life Sciences	Cat # 28907546

### Lead Contct and Materials Availability

Further information and requests for resources and reagents should be directed to, and will be fulfilled by, the lead contact Martin C Moncrieffe (mcm35@cam.ac.uk). This study did not generate new unique reagents.

### Experimental Model and Subject Details

#### Bacterial Strains

*Escherichia coli* BL21DE3 Rosetta 2 cells (Novagen) used for protein production were cultured in LB medium supplemented with the appropriate antibiotics.

#### Human HEK293T Cells

HEK293T cells were culfured in DMEM (Gibco) supplemented with 10% FCS and 100 U/ml penicillin/100 μg/ml streptomycin.

### Method Details

#### Protein Expression and Purification

The expression and purification of IRAK4^DD^ has been described previously (Motshwene et al., 2009). The N-terminal death and intermediate domains of hMyD88 (1–151) in addition to a non-cleavable C-terminal Streptactin II tag were cloned into pMCSG7 (DNASU) and expressed as an N-terminal hexahistidine-tagged fusion protein in *E*. *coli* BL21 DE3 Rosetta 2 (Novagen). Cells were grown in LB medium with shaking (220 rev/min) at 310 K to an OD_600 nm_ of 0.8 after which the temperature was reduced to 293 K and 1 mM IPTG added and protein expression continued for 12–16 hrs. Cells expressing hMyD88^DD^ were resuspended in Buffer A (20 mM Tris, 30 mM NaCl, 1 mM EDTA) supplemented with 1 ml BugBuster (Novagen) pH 8.0 and lysed using an Emulsiflex C5 (Avestin) homogeniser. The lysate was clarified by centrifugation at 99000 × g for 20 min at 277 K, applied to a 5 ml StrepTrap HP column (GE Healthcare), and washed with ten column volumes of Buffer A. The bound protein was eluted with 10 mM desthiobiotin (Sigma) in Buffer A. Pooled samples containing hMyD88^DD^ were incubated with TEV protease at 277 K to remove the hexa-histidine tag, concentrated and further purified by S200 (GE Healthcare) size exclusion chromatography using a buffer containing 20 mM Tris, 20 mM NaCl, pH 8.0 and 5% glycerol for the AUC samples.

#### Circular Dichroism Spectroscopy and Differential Scanning Fluorometry

Spectra in the farUV (195–250 nm) were recorded on an Aviv 410 spectropolarimeter. Protein samples (35 μM) were in 20 mM Tris, 20 mM NaCl, 5% glycerol pH 7.5. The instrument bandwidth was 1 nm, the wavelength scan increment 0.5 nm and the averaging time 1 s. Three spectra were accumulated and averaged. Continuous temperature scans at fixed wavelength (222 nm) were performed in the range 283—368 K with a temperature step of 1 K. Differential scanning fluorometry experiments were performed using Spyro Orange (Thermo Fisher) as the fluorescent probe and a RotorGene 6000 (Qiagen) thermocycler.

#### Analytical Ultracentrifugation

Analytical sedimentation velocity and equilibrium measurements were performed using a Beckman XLA ultracentrifuge at 293 K and a sample concentration of 35 μM. For sedimentation velocity measurements, the sample and reference volumes were both 400 μl and data were acquired at 120 s intervals at rotor speeds of 30,000 rev/min using an An60Ti rotor (Beckman Coulter). Buffer density and viscosity were estimated using SEDINTERP (Laue et al., 1992) and the sedimentation profiles were analysed with SEDFIT (Schuck, 2000). Sedimentation equilibrium experiments were conducted using 180 μl sample volume. Absorbance data was acquired at 250 and 280 nm and rotor speeds of 3200 and 4200 rev/min. The attainment of equilibrium was determined by subtraction of successive scans until the root mean square difference was constant. Global analysis of the equilibrium data was performed using SEDPHAT (Schuck, 2003).

#### CryoEM Reconstruction

A 4 μl sample of hMyD88^DD^ at 1 mg/ml was applied to a glow discharged Quantifoil R1.2/1.3 holey carbon grid (Quantifoil). Grids were blotted and frozen in liquid ethane using a Vitrobot Mark IV (FEI) instrument (277 K, blot force 0, blot time 3 s, humidity 100%) and stored in liquid nitrogen. Grids containing vitrified samples were imaged using a Titan Krios (FEI) microscope operating at 300 kV and with a Falcon III (FEI) direct electron detector in counting mode. Movies were recorded using a nominal magnification of 75, 000 ± 1500× and a calibrated pixel size of 1.06 Å/pixel. During data collection, 48 frames were acquired with a total dose of 44.1 e⋅Å^-2^ corresponding to a per frame dose of 0.92 e⋅Å^-2^ An initial estimate of the helical symmetry was obtained using SPHIRE (Moriya et al., 2017). Filaments were selected from 200 micrographs using *sxhelixboxer*.*py* and segments were windowed using a box size of 220 × 220 pixels with 10 pixels overlap between them. The averaged power spectrum of all aligned patches was then calculated which revealed a pattern of layer lines typical of objects with helical symmetry (Figures S3A and S3B) having a meridional reflection at 0.161 Å^-1^ implying that the axial rise per subunit is approximately 6.2 Å. The azimuthal rotation per subunit obtained by indexing was 98.2°. Subsequent calculations were performed in RELION3.0 (Scheres, 2012). Movie stacks were motion corrected using MotionCor2 (Zheng et al., 2017) and contrast transfer function parameters were estimated with Gctf1.06 (Zhang, 2016) using an amplitude contrast of 0.1. Micrographs that had large defocus values relative to the nominal values set at the microscope were not used in subsequent processing. The range of defocus values used was −0.9 μm to −3.2 μm. Parameters for auto-picking filaments in RELION were optimised using twenty-five micrographs having defocus values from the range given above. Segments were extracted using a box size of 220 pixels and an overlap of 7 pixels yielding 1,257,342 overlapping helical segments from all micrographs. Reference-free 2D classification using a regularisation value of T=2 was then performed to identify homogeneous subsets. The selected class averages contained 1,229,488 overlapping helical segments. An initial model was generated using the Stochastic Gradient Descent algorithm (Punjani et al., 2017) as implemented in RELION. High resolution refinement using all 1,229,488 overlapping segments was performed during which the helical parameters were refined using 3D auto-refinement in RELION. The refined helical symmetry values were 5.98 Å (rise) and 98.01° (azimuthal rotation per subunit). A resolution estimate of 3.1 Å was obtained using the Fourier shell correlation at 0.143 between two independently refined half-maps. The map was sharpened using the post-processing routines in RELION and a soft mask (Figure S3E.) resulting in a *B*-factor of −150 Å^2^. MOLREP (Vagin and Isupov, 2001) was used to place a single copy of the hMyD88^DD^ monomer taken from the crystal structure of the heterooligomeric hMyD88 ^DD^ -IRAK4 ^DD^ -IRAK2 ^DD^ complex (PDB code, 3MOP) (Lin et al., 2010) into the post-processed cryoEM map. Several rounds of real-space refinement in PHENIX (Adams et al., 2010, Wang et al., 2014) followed by model building in COOT (Emsley et al., 2010) were then performed. MOLREP (Vagin and Isupov, 2001) was then used to place thirteen copies of the extended hMyD88 ^DD^ model into the density followed by real space refinement as before. A comparison of the reconstructed map and the PDB model in Chimera (Pettersen et al., 2004) reveals that the pixel size at the specimen level was 1.065 Å/pixel (Figure S3D). Model validation was performed using PHENIX (Adams et al., 2010). Figures containing CryoEM maps and PDB models were made using UCSF ChimeraX (Goddard et al., 2018).

#### Dynamic Light Scattering

DLS measurements were performed at 298 K using a Zetasizer Nano S (Malvern Panalytical) instrument and a Hellma quartz cuvette with a 3 mm path length and centre 9.65 mm. All protein samples were passed through 20 μm filters prior to use. 20 μl of MyD88^DD^ (45 μM) was placed into the cuvette and aliquots of 0.8 mM IRAK4^DD^ added so that the total volume in the cuvette did not exceed 26 μl. All measurements were performed at 298 K and analysed using the continuous hydrodynamic radius distribution model in SEDFIT(Schuck, 2000).

#### Cell Culture

The generation of murine immortalised bone marrow derived macrophage (iBMDM) cells expressing GFPMyD88 from MyD88/iBMDMs has been described (Latty et al., 2018). HEK293T cells were seeded at approximately 50% confluency into 12-well plates, transfected using a 3:1 (μl:μg) ratio of Genejuice (Novagen) transfection reagent and incubated for 96 h at 310 K and 5% CO_2_. A total of 1.5 μg of plasmid DNA (0.5 μg each of p891, pMDG and pHR MyD88GFP) was used per well. The supernatant containing lentiviral particles was centrifuged for 5 min at 1200 rev/min, diluted (1:64) using complete DMEM medium and added to MyD88/iBMDMs for 24 h at 310 K and 5% CO_2_. The medium was then replaced with complete DMEM and iBMDMs grown for an additional 24 h.

#### Microscopy

Details of the experimental procedures used for acquiring TIRFM and LSFM images have been published (Ponjavic et al., 2018). A 15 mW fibre-coupled 488 nm diode laser (iFLEX2000,Qioptic) and an excitation filter (FF01488/625, Semrock) was used. Light enters the microscope (Eclipse TiU, Nikon) through a 100×, 1.49 numerical aperture (NA) oil immersion objective lens (MRD01991, Nikon) and is focused at the back focal plane of the objective lens. The internal magnification of the microscope was used to achieve a final magnification of 150×. The fluorescence emission was bandpass filtered (67031,Edmund Optics) and focused on to an EMCCD camera (Evolve 512 Delta, Photometrics). Coverslips were plasma cleaned (Harrick Plasma) in an Argon atmosphere for 30 min and coated with solutions of 0.01% (w/v) poly-L-lysine 150-300 kDa (Sigma-Aldrich). The coated coverslips were then washed three times in PBS. 40 μl aliquot of cells (25000 cells/ml) was added to the coated coverslips and stimulated when required by the addition of 2 μl of 100 ng/ml LPS. Two cylindrical lens were used to generate a wide sheet of light and a perpendicular secondary objective lens (10× the working distance of 33.5 mm) and having a numerical aperture of 0.2 (Mitutoyo) was used to produce the excitation onto the samples. The laser beam, acquisition setup and parameters were the same as for TIRF. The sample chamber was constructed by attaching two plasma clean coverslips at an 85° angle using a putty-like pressure-sensitive adhesive. Photobleaching was performed on cells fixed with paraformaldehyde and 0.2% glutaraldehyde. Image stacks using TIRF were acquired for 100 frames and ImageJ was used for data analysis as described (Latty et al., 2018).

### Quantification and Statistical Analysis

Intensity distributions for TIRFM, Light-sheet microscopy and photobleaching were obtained using ImageJ software package.

### Data and Code Availability

Cryo-EM maps and atomic coordinates are deposited in the Electron Microscopy Data Bank (EMD4405) and Protein Data Bank (PDB 613N) respectively.

## Supplemental Information

Document S1. Figures S1–S6 and Table S1

Document S2. Article plus Supplemental Information
