# Supplementary material for: MyD88 Death-Domain Oligomerization Determines Myddosome Architecture: Implications for Toll-like Receptor Signaling
Source: Structure. 2020 Mar 3;28(3):281–289.e3. doi: 10.1016/j.str.2020.01.003 (PMC7054835; doi:10.1016/j.str.2020.01.003)
Supplement: Document S1. Figures S1–S6 and Table S1 [file mmc1.pdf]

Structure, Volume *28*

Supplemental Information

MyD88 Death-Domain Oligomerization Determines Myddosome Architecture: Implications for Toll-like Receptor Signaling

Martin C. Moncrieffe, Daniel Bollschweiler, Bing Li, Pawel A. Penczek, Lee Hopkins, Clare E. Bryant, David Klenerman, and Nicholas J. Gay

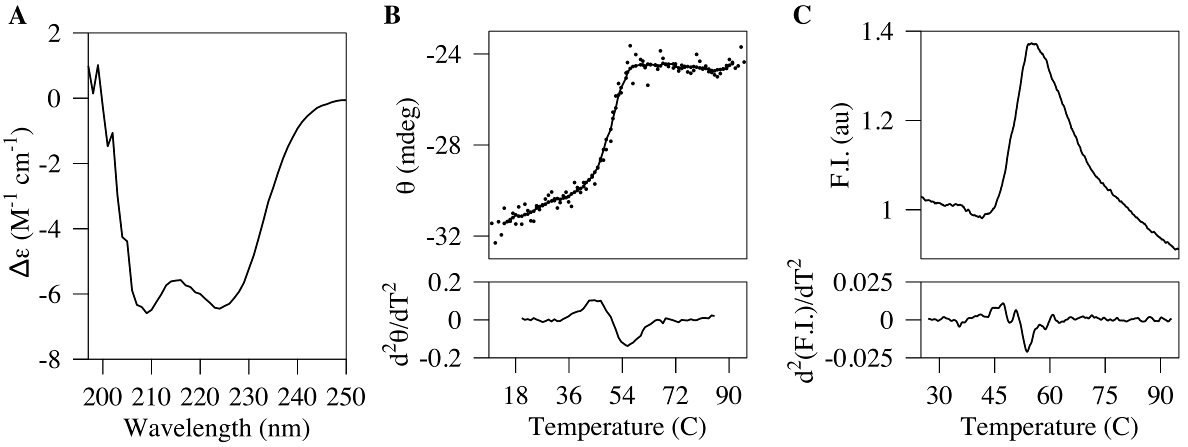

Figure S1. Characterization of the hMyD88^{DD} oligomer. Related to Figure 1 and Figure 2 and STAR Methods section 'Circular dichroism spectroscopy and differential scanning fluorometry' (**A**) The far-UV circular dichroism spectrum of the oligomeric MyD88^{DD} displays characteristic peaks reflective of α-helical secondary structure at 209 nm 223 nm respectively. Thermal denaturation profiles measured by monitoring the decrease of ordered secondary structure content using circular dichroism (**B**) in the far-UV (222 nm) and the fluorescence of SPYRO orange (**C**) gives T_m values of 55.7 °C and 53.4 °C.

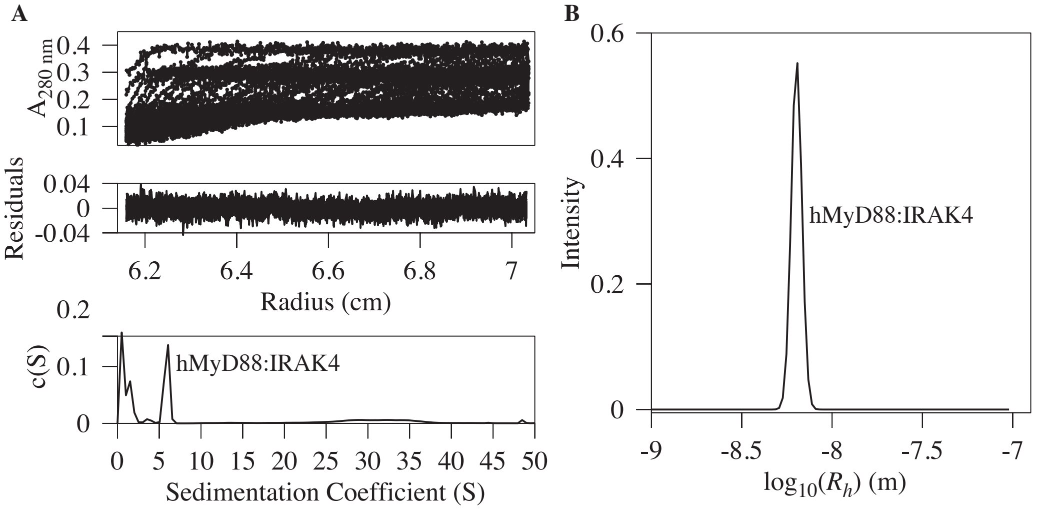

Figure S2. MyD88^{DD} filaments interact with IRAK4^{DD}. Related to Figure 4. (**A**) Formation of the hMyD88^{DD}-IRAK4^{DD} complex from helical hMyD88^{DD} filaments. (**B**) Dynamic light scattering distribution of the hMyD88^{DD}-IRAK4^{DD} complex purified by S200 size exclusion chromatography.

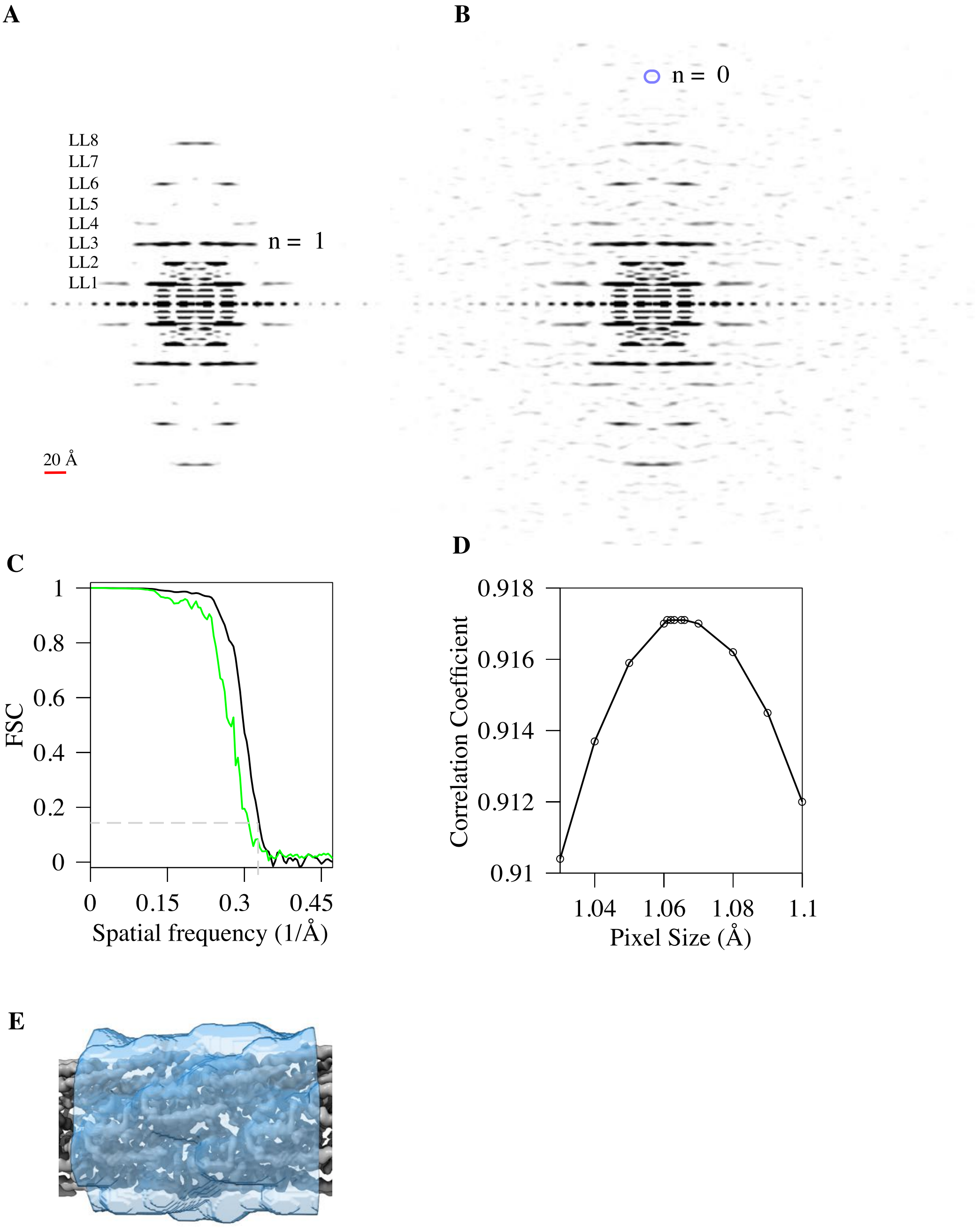

Figure S3. Reconstruction of the MyD88^{DD} filament. Related to Figure 2 and Figure 3. Averaged power spectrum of hMyD88^{DD} filaments display layer lines (LL) which are typical of objects possessing helical symmetry. (**A**) The first eight layer lines (LL1–LL8) are shown and the Bessel order ($n = 1$) for LL3. (**B**) The first meridional line for which the Bessel order is zero ($n = 0$), which represents the helical rise is shown circled in blue. (**C**) Fourier shell correlation. Masked (black), unmasked (green). The dotted line depicts the point where FSC = 0.143 and the corresponding resolution. (**D**) Pixel size at the specimen level. Plot of the correlation coefficient for the fit between the three-dimensional coordinates of MyD88^{DD} (PDB code 3MOP) and the cryo-EM map as a function of voxel size. (**E**) Reconstructed hMyD88^{DD} filament and the soft mask used in post-processing.

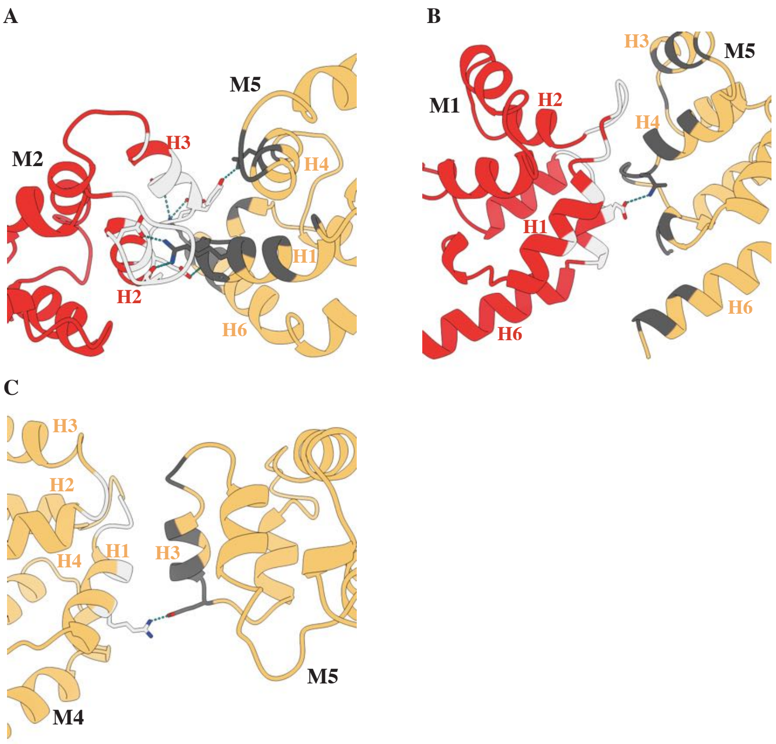

Figure S4. Interfaces of the MyD88^{DD} filament. Related to Figure 3. Type I (**A**), II (**B**), and III (**C**), interfaces seen in the hMyD88^{DD} filament. The respective *a* or *b* interaction surfaces are displayed in dark- and light-grey respectively. Details of the actual residues involved are given in Table S1.

Table S1. Typical interfacial residues in the MyD88^{DD} filament. Related to Figure 3. Residues in the type I, II and III interface of the MyD88 death domain filament. Residues involved in hydrogen bonds and salt-bridges are indicated by H and S respectively.

Type I		Type II		Type III	
**a**	**b**	**a**	**b**	**a**	**b**
A23	R40	S19	R32	E57^{S}	R32^{S}
L25	T41^{H}	L20	F36	Y58	L35
N26^{H}	Q42	P21	V39	L59	F3
M27	V43	A23	T41	E60	N38
R28^{H}	A44^{H}	F56	Q42	R62	V39
R30	A45	E60	V43	Q63	R40
R31^{H,S}	D46^{S}	Q63	A44	T66	A68
S34	T48^{H}	L64	A45	Q67	D69
L35	A49	Q67	L96		E104
N38	Q52^{H}	R73	G97		
A68	D55	D76	R98		
D69	F56	A77	D99		
R73	E57^{S}	Q79^{S}	D100^{S}		
D76^{H,S}	Y58^{H}	G80	L103		
Q79	L59	R81	E104^{H}		
K115	E60	P82			
Y116	I61	Y116^{H}			
K119^{S}	R62^{S}	K119			
	E65	Q120			

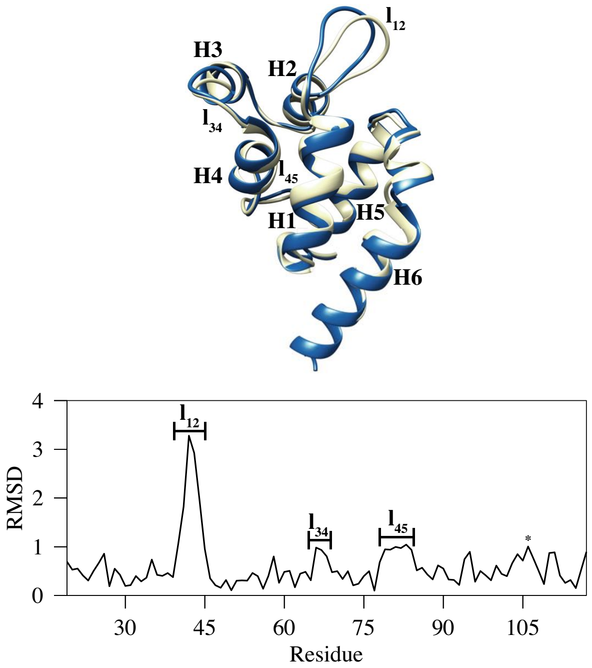

Figure S5. Superposition of MyD88^{DD} determined by cryo-EM and X-ray crystallography. Related to Figure 3. Structure of MyD88^{DD} from the death domain filament (blue) and the ternary MyD88-IRAK4-IRAK2 Myddosome (yellow). The r.m.s.d. across all atom pairs is 0.76 Å. The largest differences occur in the loop regions connecting the α-helices H1–H2 (l_{12}), H3–H4 (l_{34}), H4–H5 (l_{45}) and a break in H6 at G106($*$).

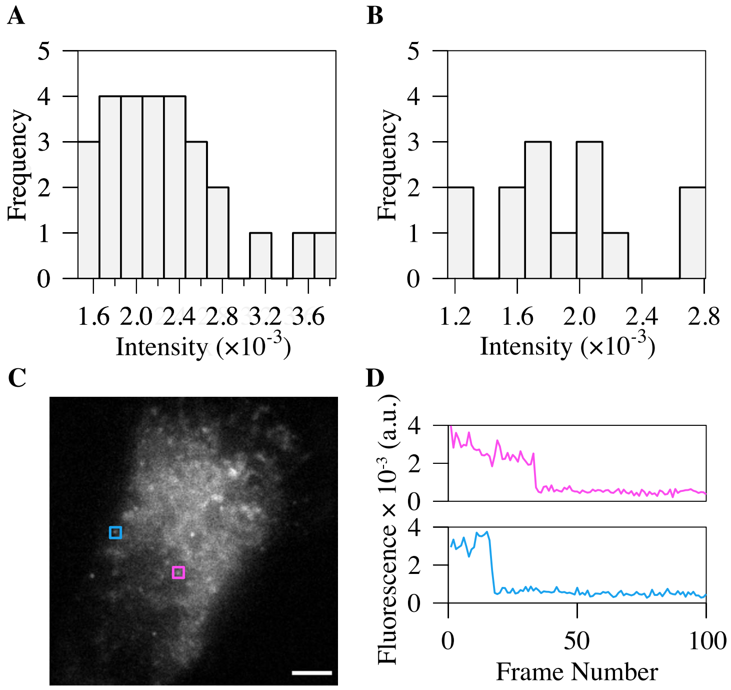

Figure S6. MyDDosome formation in cells. Related to Figure 5. Histograms of the intensity distributions for GFP-MyD88 in unstimulated (**A**) and stimulated (**B**) virally transduced MyD88$^{-/-}$ bone derived macrophage macrophages. Determination of monomeric MyD88 intensity by photobleaching. The locations indicated by coloured squares (**C**) were bleached and the intensity trajectory after bleaching is shown (**D**). At these locations, GFP-MyD88 bleaches in a single step suggesting that the protein is monomeric. The length of the scale bar is 5 μm.
